# Spontaneous gaseous necrotizing myofasciitis in a patient with B-cell lymphoma status-post induction chemotherapy

**DOI:** 10.1093/jscr/rjaf910

**Published:** 2025-11-20

**Authors:** Margaret Shields, Mason H Remondelli, Amy M Wang, Natalia K Barzanji, Jonathan Wang, Emad S Madha, Matthew J Bradley, Patrick F Walker, John C Graybill

**Affiliations:** Department of Surgery, The Walter Reed National Military Medical Center, 901 Rockville Pike, Bethesda, MD 20889, United States; Department of Surgery, The Walter Reed National Military Medical Center, 901 Rockville Pike, Bethesda, MD 20889, United States; Department of Surgery, The Walter Reed National Military Medical Center, 901 Rockville Pike, Bethesda, MD 20889, United States; Department of Surgery, The Walter Reed National Military Medical Center, 901 Rockville Pike, Bethesda, MD 20889, United States; Department of Surgery, The Walter Reed National Military Medical Center, 901 Rockville Pike, Bethesda, MD 20889, United States; Department of Surgery, The Walter Reed National Military Medical Center, 901 Rockville Pike, Bethesda, MD 20889, United States; Department of Surgery, The Walter Reed National Military Medical Center, 901 Rockville Pike, Bethesda, MD 20889, United States; Department of Surgery, The Uniformed Services University of the Health Sciences, 4301 Jones Bridge Rd, Bethesda, MD 20814, United States; Department of Surgery, The Walter Reed National Military Medical Center, 901 Rockville Pike, Bethesda, MD 20889, United States; Department of Surgery, The Uniformed Services University of the Health Sciences, 4301 Jones Bridge Rd, Bethesda, MD 20814, United States; Department of Surgery, The Walter Reed National Military Medical Center, 901 Rockville Pike, Bethesda, MD 20889, United States; Department of Surgery, The Uniformed Services University of the Health Sciences, 4301 Jones Bridge Rd, Bethesda, MD 20814, United States

**Keywords:** Myonecrosis, necrotizing fasciitis, necrotizing soft tissue infection, Polymicrobial infection, immunosuppression, sepsis, shock

## Abstract

A 50-year-old female with diffuse large B-cell lymphoma and recent completion of chemotherapy with Pola-R-CHP presented to the emergency department with right lower extremity weakness and pain, hypotension, tachycardia, and altered mental status, along with tense, edematous, and pulseless compartments of the right lower extremity. The patient was taken emergently to the operating room with trauma and orthopedic surgery and found to have a severe necrotizing infection involving the muscles of the right lower extremity, ascending to the retroperitoneum, involving the right iliacus muscle, right iliopsoas, and ascending colon. At that time, she underwent a right transfemoral amputation, a right hemicolectomy, and was left in discontinuity to stabilize her in the ICU until returning the next day. NSTIs (necrotizing soft tissue infection) are rare but rapidly progressive, life-threatening infections. This case report highlights the importance of early and aggressive surgical intervention to improve the survival of patients with NSTIs.

## Introduction

Necrotizing soft tissue infections (NSTI) are rare but rapidly progressive, life-threatening infections characterized by necrosis of the subcutaneous tissue, fascia, and, in some cases, muscle. The diagnosis of NSTI is often clinical, manifesting hallmark signs and symptoms, which include erythema, crepitus, pain out of proportion, and systemic signs of illness with leukocytosis, fever, and tachycardia. The extent of necrosis can be divided into three zones: (i) complete necrosis, (ii) partially viable and may be salvageable with careful debridement, (iii) uninfected, healthy tissue. Frankly necrotic tissue is evident by the lack of bleeding during surgical debridement, and compromised fascial integrity. A delay in diagnosis or misdiagnosis can lead to poor outcomes, with mortality rates ranging from 20% to 80% [1]. Because of this, prompt recognition and aggressive management, including early surgical debridement, are critical to improving patient survival [2].

**Figure 1:**
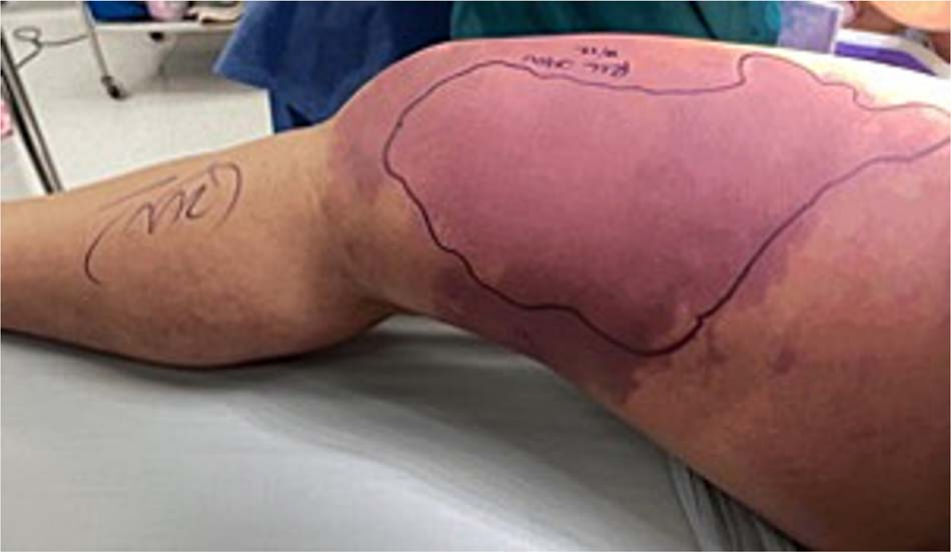
Right medial leg.

Furthermore, NSTIs are frequently associated with immunocompromised states, including diabetes, chronic liver disease, and hematologic malignancies. Among hematologic conditions, B-cell lymphomas are noteworthy due to their potential to manifest with cutaneous involvement and paraneoplastic phenomena, complicating the clinical picture [3]. The case report underscores the need for prompt intervention with a multi-disciplinary approach in managing complex presentations of NSTIs associated with hematologic malignancies.

## Case report

**Figure 2:**
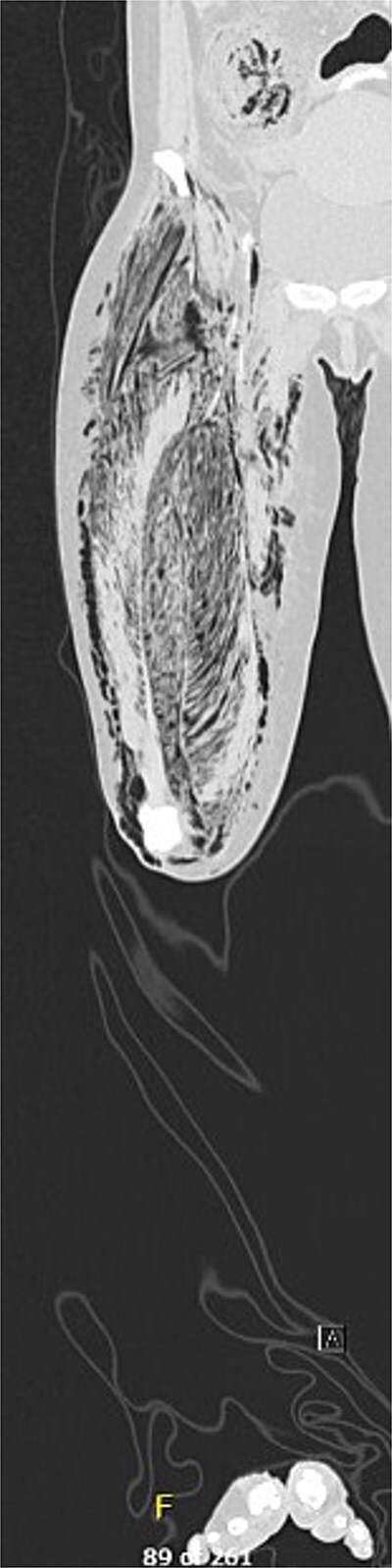
CTA right lower extremity, coronal.

A 50-year-old female with a history of biopsy-proven diffuse large B-cell lymphoma status post induction chemotherapy with Pola-R-CHP (polatuzumab, rituximab, cyclophosphamide, doxorubicin, and prednisone) presented to the emergency department with right lower extremity (RLE) weakness and pain, hypotension, tachycardia, and altered mental status. Physical exam revealed a cool, tense, edematous RLE, with absent distal pulses and no Doppler signals (Fig. 1). Motor strength was severely diminished. Labs were notable for a WBC 1.2 × 10^3^/mcl, hemoglobin 7.6 g/dl, CO_2_ 17 mmol/L, AGAP 24 mmol/L, creatinine 1.73 mg/dl, CRP 35.6 mg/dl, and a LRINEC score of 10. An X-ray of the RLE demonstrated extensive air dissecting superficial, deep, and intramuscular fascial planes. Prior to consultation, a CT (Fig. 2) obtained in the emergency department, demonstrated extensive necrosis of the right thigh, with gas tracking from the iliac vein down to the popliteal vein, into the femoral intramedullary space, and the retroperitoneum. Additionally, diffuse fat stranding and pneumatosis intestinalis were noted in the cecum and terminal ileum.

The patient was diagnosed with an NSTI of the RLE, with extensive myonecrosis extending into the retroperitoneum. Broad-spectrum antibiotics (vancomycin, cefepime, and metronidazole) were initiated. In the emergency room, the patient’s condition continued to rapidly deteriorate with worsening skin changes, progressive sensory loss in the RLE, and escalating vasopressor requirements. She was taken emergently to the operating room for an exploratory laparotomy and debridement of the RLE.

Surgical exploration revealed extensive necrotizing infection of the RLE with widespread muscle necrosis and devitalization. The infection appeared to extend into the retroperitoneum, with involvement of the right iliacus and iliopsoas muscles. A right transfemoral amputation was performed due to extensive myonecrosis involving the majority of the right thigh and lower gluteal musculature. The cecum and right ovary were necrotic without gross cecal perforation. The gallbladder, liver capsule, and uterus appeared inflamed. A right hemicolectomy was performed, and the bowel was left in discontinuity with temporary abdominal closure. The patient was then transferred to the Surgical Intensive Care Unit, intubated, and in critical condition. Blood and tissue cultures returned rapidly positive for gram-positive rods, prompting Infectious Disease consultation and initiation of meropenem and linezolid for suspected Clostridium septicum bacteremia.

**Figure 3:**
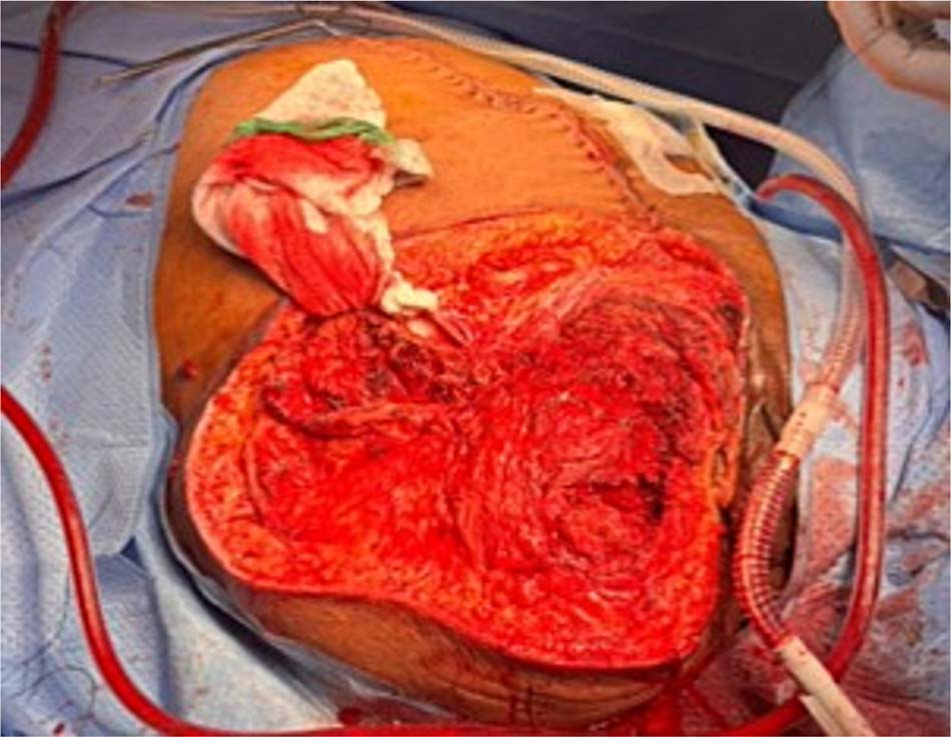
Intra-operative right hip disarticulation.

Post-operatively, the patient developed new-onset, progressive erythema and induration of the left lower extremity (LLE). Repeat CT imaging demonstrated NSTI involving the mesentery and retroperitoneum, with marked edema of intestinal and gallbladder walls. New findings included NSTI of LLE involving all tissue planes, most severe in the left gluteal musculature and thigh, and necrosis of all tissue planes of the residual RLE and pelvis. The patient was taken back to the operating room for right hip disarticulation, debridement of the LLE, and abdominal re-exploration (Fig. 3). The patient also underwent bilateral salpingo-oophorectomy and cholecystectomy given the ischemic-appearing nature of both organs. While the patient continued to improve, after a multidisciplinary discussion, an end ileostomy with abdominal closure was eventually performed due to ongoing vasopressor requirement. She was eventually extubated, weaned off sedation, and downgraded to the surgical ward. The patient ultimately recovered well throughout the remainder of her hospital stay.

## Discussion

NSTIs are a rare but rapidly progressive, life-threatening infection characterized by necrosis of subcutaneous tissue, fascia, and sometimes muscle, often accompanied by severe systemic toxicity and septic shock, as demonstrated by this case report. NSTIs can present hyperacutely, as seen in our patient, rapidly progressing to multiorgan failure, or more subacutely, with a slower disease course [4]. The most common causes of NSTIs are related to trauma, diabetes, chronic wound infections, obesity, alcoholism, chronic liver disease, and immunosuppression [5]. In our case, the patient was likely more susceptible to a necrotizing infection due to immunosuppression from chemotherapy, and may have initially seeded the infection from a bone marrow biopsy performed a few weeks earlier, potentially from colonic perforation by the biopsy needle, allowing *C. septicum*, a known colonic flora, to become walled off in the retroperitoneum. Higher rates of lower extremity infections have been reported, varying between 32% and 58%, although infection of the upper extremity portends a higher mortality rate [6].

In early stages, NSTIs, can be challenging to diagnose, as its clinical appearance may resemble cellulitis, and patients may not yet exhibit signs of septic shock. In patients with hematologic malignancies, not only are they at a higher risk for infections, but it can be even more difficult to elucidate a potential infection from a paraneoplastic syndrome associated with their malignancy. As a result, it is critical to have a high degree of suspicion [7]. In a study by Rouse *et al*., 27 patients with NSTIs were studied. All but four were polymicrobial, with an overall mortality rate of 73%. Two of 15 patients who underwent >1 debridement survived, and 11 of 12 patients with delayed treatment (>12 h) died. A high index of suspicion, prompt recognition, and rapid surgical debridement of NSTIs are essential for survival [8]. Ultimately, it is a surgical emergency that requires rapid debridement, excision of nonviable tissue, and broad-spectrum intravenous antibiotics.

## Conclusion

This case highlights the interplay between NSTI and underlying hematologic malignancy, illustrating the diagnostic and therapeutic challenges encountered. It also underscores the need for heightened clinical suspicion and prompt multi-disciplinary intervention in NSTI management and treatment.
